# Clinicopathological and immunohistochemical study of 29 cases of solid-pseudopapillary neoplasms of the pancreas in patients under 20 years of age along with detailed review of literature

**DOI:** 10.1186/s13000-020-01058-z

**Published:** 2020-12-09

**Authors:** Nasir Ud Din, Shabina Rahim, Jamshid Abdul-Ghafar, Arsalan Ahmed, Zubair Ahmad

**Affiliations:** 1 Department of Pathology and Laboratory Medicine, Aga Khan University Hospital, Karachi, Pakistan; 2 Department of Pathology and Clinical Laboratory, French Medical Institute for Mothers and Children (FMIC), Kabul, Afghanistan

**Keywords:** Pancreas, Solid pseudopapillary neoplasm (SPN), Young females, Low-grade malignant tumor, Excellent prognosis, TFE3, Progesterone receptor

## Abstract

**Background:**

Pancreatic Solid Pseudopapillary Neoplasms (SPNs) are rare low-grade malignant tumors with a marked preponderance for young females. Objective was to describe the morphology, differential diagnosis, and prognosis of SPNs in patients under 20 years of age and present a detailed review of literature.

**Methods:**

A total of 29 cases in patients under 20 years of age reported as SPN during the period January 2014 to December 2019, were included in the study. These included 19 resection specimens, 4 incision biopsies and 6 cases received as blocks for second opinion. Hematoxylin and eosin (H&E) slides as well as immunohistochemistry (IHC) slides of all cases were retrieved and reviewed by the authors. TFE3 and Progesterone Receptor were performed retrospectively.

**Results:**

Twenty-eight of the 29 patients were females. Ages of patients ranged from 12 to 19 years. Nineteen cases were resections. Tail was the commonest location. Mean tumor size was 9.5 cm. In 89.5% cases, tumor was confined to the pancreas. In 2 cases, distant metastasis was present. In 2 cases, extension beyond pancreas was seen. Solid and pseudopapillary areas were seen in all cases while other features were variable. Beta catenin and Cyclin D1 were positive in most cases while TFE3 was positive in 57% cases. Progesterone Receptor (PR) was positive in all 13 cases in which it was performed. Follow up was available in 14 patients. Follow up period ranged from 3 to 70 months. Twelve were alive and well without recurrence or metastasis while 2 were alive with recurrence and metastasis to liver and omentum respectively.

**Conclusions:**

Although many studies on SPNs have been published, surgeons, oncologists and even pathologists in this part of the world are often not aware of these rare tumors leading to inaccuracies and delays in diagnosis. In addition, this paper focusses on the interesting observation that the majority of SPNs diagnosed in our department during study period occurred in patients under 20 years of age (29 versus 21 in patients over 20). However, clinico-epidemiological, morphologic and prognostic features were similar in both age groups. Possibility of SPNs should always be considered in case of pancreatic neoplasms occurring in patients under 20 years of age as well. We believe that this is a very interesting and helpful study for the clinicians as well as the pathologists.

## Background

Solid pseudopapillary neoplasms (SPNs) of the pancreas are defined by the new 5th edition of the World Health Organization (WHO) Classification of Digestive System Tumors as low grade malignant tumors composed of poorly cohesive epithelial cells which form solid and pseudopapillary structures and lack a specific line of pancreatic epithelial differentiation [1]. SPNs were first described by Frantz in 1959 as pancreatic papillary cystic tumors [2]. WHO first classified SPNs in 1996 as solid pseudopapillary tumors and re-classified them in 2010 as SPNs [3, 4]. SPNs are rare tumors comprising only 1 to 3% of all pancreatic tumors and show a marked female preponderance and excellent prognosis [5]. Various studies have reported mean age at diagnosis ranging from 24 to 39 years and age range of 7 to 83 years [6–18]. Cases in children, older patients and males have also been reported [19]. SPNs grow slowly and may become considerably large before they cause symptoms. Some patients may present with ill defined, mild upper abdominal pain, while others are asymptomatic. SPNs usually come to light on abdominal ultrasound or Computed Tomography (CT) scan performed in patients with persistent, long standing, unexplained upper abdominal pain. At other times, SPNs are discovered incidentally during imaging studies performed for some other reason. Owing to their often-silent nature, they may be quite bulky when first discovered [1, 20]. SPNs do not show preference for any specific part of the pancreas [9]. In Wang et al’s series, 38.1% cases were located in the head of pancreas while 49.5% were localized to the body and tail [8]. This predilection for the head or tail was also noted in several other studies [12, 14, 21]. Grossly, SPNs are usually solitary [15], encapsulated and typically sharply demarcated from adjacent non neoplastic pancreatic tissue. Surgical removal is usually easy, and enucleation of the tumor is often performed especially in tumors smaller than 5 cm in size [8, 12]. All resected tumors in our study were nodular and circumscribed and partly or wholly encapsulated. Various studies have reported wide variations in tumor size ranging from 1.5 cm to 22 cm. However, malignancy in SPNs does not correlate with tumor size [22]. Mean tumor size has ranged from 4.7 to 9.5 cm in various studies [5, 8, 9, 12, 14, 16, 18, 23]. Tumor size in our resection specimens ranged from 3 to 14 cm with mean size of 9.5 cm. Tumors located in the distal body and tail tend to be larger [24]. Apart from enucleation, distal pancreatectomy and pancreaticoduodenectomy (Whipple’s resection) are also commonly used to treat SPNs [11, 14, 16, 25]. More than 90% patients undergo primary tumor resection [11, 12]. Histological and/or cytological evaluation remain the gold standard in reaching a definitive diagnosis [26]. Histologically, SPNs show solid sheets of tumor cells along with areas showing cells oriented around delicate fibrovascular cores [1]. Periodic acid Schiff (PAS) positive hyaline globules constitute a common and typical feature. Mitoses are usually rare, atypical mitoses are not seen and MIB-1 (KI-67) proliferative index is very low [5]. Histologically, the majority of SPNs run a benign course and 5-year survival rates are excellent [5, 12–15]. We have diagnosed a number of these tumors over the last two decades. However, in recent years we have observed in our practice that the majority of SPNs were reported in patients younger than 20 years of age. The aim of the present study was to describe the clinico-epidemiological as well as morphologic and immunohistochemical (IHC) findings and behavior of SPNs diagnosed in our practice. We also aim to present a detailed review of published literature regarding the histogenesis, clinico-pathological features with emphasis on newer IHC antibodies such as Transcription Factor E3 (TFE3), differential diagnostic considerations, therapeutic strategies, prognosis and biological behavior including the likelihood of malignancy and the factors which may be important in determining aggressive behavior in these tumors based on published literature.

## Materials and methods

A total of 29 cases in patients under 20 years of age reported as SPN of pancreas reported in the Section of Histopathology, Department of Pathology and Laboratory Medicine, Aga Khan University Hospital Karachi during the period January 2014 to December 2019 were included in the study. These included 19 resection specimens, 4 incisional biopsies and 6 cases received as blocks for second opinion.

### Preparation of tissue samples

Tru cut and incisional biopsies as well as resections were fixed in 10% buffered formalin. All tru cut and incisional biopsies were entirely submitted for histological examination. In case of resections (distal pancreatectomy, pancreaticoduodenectomy or Whipple Resection), multiple representative sections were submitted, as per established protocols, from the tumor, adjacent areas, resection margins and lymph nodes (if present).

### Pathological analysis

In all cases the initial diagnosis made by the primary pathologist (to whom case was originally assigned) was reviewed, considering the rarity of these tumors, by one or more pathologists with special interest and expertise in gastrointestinal, biliary and pancreatic pathology (ND and ZA, the senior authors of this paper) before the case was finally signed out.

### IHC staining

Primary and reviewing pathologists performed a number of IHC stains to complement the histological diagnosis and to eliminate close histological mimics. IHC stains commonly performed included CD10, CD56, beta catenin, Cyclin D1, CD99, Cytokeratins, Chromogranin A, Synaptophysin and Progesterone Receptor (PR). Since acquiring TFE3, this antibody was also commonly performed. The large IHC panel helped in reaching an accurate diagnosis.

Hematoxylin and eosin (H&E) slides as well as IHC slides of all cases were retrieved and reviewed by the authors. The two senior authors (ND & ZA) who have special interest and expertise in Gastrointestinal, biliary tract and pancreatic pathology, reviewed the histologic and IHC features of all 29 cases.

### Definitions of histological features

Solid tumor component in SPNs was defined as being composed of poorly cohesive monomorphic cells that cling to hyalinized or myxoid fibrovascular cords. Pseudopapillae in SPNs are formed when the neoplastic cells detach from fibrovascular stalks. Hyaline globules in SPNs are defined as intracytoplasmic PAS positive round eosinophilic bodies. These constitute a common, although nonspecific histological feature which can be useful in formulating a differential diagnosis in these tumors. Nuclear grooves in SPNs are defined as longitudinal invaginations or indentations of the nuclear envelope bilayer and are another common histologic feature of these tumors. Clear cells in SPNs are defined as cells with abundant clear cytoplasm. SPNs composed predominantly of clear cells are termed ‘clear cell variant’. Clear cells are multivacuolated, do not contain glycogen, lipid or mucin and seem to be formed as a result of dilatation of endoplasmic reticulum and mitochondria. Cystic degeneration involving < 5% of the tumor is common in SPNs. SPNs with greater than 5% cystic degeneration are termed the “microcystic variant”. Large atypical pleomorphic cells and multinucleated tumor giant cells (with multiple, enlarged hyperchromatic irregular nuclei and abundant eosinophilic cytoplasm) are typically present in the solid areas of SPN in a background of monomorphic cells. They probably represent degenerative changes in tumor cells and do not appear to affect the prognosis.

### IHC preparation

In IHC, special emphasis was placed on expression of TFE3 (Cell Marque anti - TFE3, MRQ-37, rabbit monoclonal primary antibody, Rocklin, CA 95677 USA), CyclinD-1 (FLEX Monoclonal Rabbit Anti-Human Cyclin- D1 clone EP12, ready to use, Dako Denmark, Glostrup, Denmark, Deko North America, Carpinteria, California USA), CD56 (FLEX Monoclonal Mouse Anti – Human CD56 clone 123C3 ready to use Dako Denmark, Glostrup, Denmark), beta catenin (FLEX Monoclonal Mouse Anti-Human beta Catenin Clone beta catenin-1, ready to use, Dako Denmark, Glostrup, Denmark), PR (FLEX Monoclonal Mouse Anti-Human Progesterone Receptor Clone PgR 636 ready to use, Dako Denmark, Glostrup, Denmark, Dako North America, Carpinteria, California USA), and CD99 (FLEX Monoclonal Mouse Anti-Human CD99, MK2 Gene Product Ewing’s Sarcoma Marker Clone 12E7 ready to use, Dako Denmark, Glostrup, Denmark) by tumor cells. For IHC testing, envision flex Immunohistochemistry method was used. Envision Flex peroxidase bleaching reagent was applied against the slide for 5 min after which slides were washed with wash buffer. Primary antibody) was then applied to the tissue for 25 to 30 min and washed again with wash buffer. Then Envision Flex / HRP (Secondary antibody was applied to the tissue for 25 to 30 min and washed with wash buffer. Following this, Envision Flex DAB + chromogen diluted in Envision flex substrate buffer was applied for 5 to 10 min and washed with wash buffer. Slides were then counterstained with Hematoxylin, washed with buffer and distilled water. Slides were then dehydrated (alcohol to xylene) and mounted in cover slipper.

### Histological features

Histological features including presence of pseudo papillary architecture, microcystic change, presence of clear cells, hyaline globules, nuclear grooves, eosinophilic cytoplasm, myxoid stroma, atypical cells and tumor giant cells, mitotic activity, calcification, cholesterol clefts, fibrosis, hemorrhage, infarction and tumor necrosis were carefully noted.

### Statistical analysis

Clinical data including age, gender, specific tumor location in pancreas, size of tumor, type of surgery, lymph node status and clinical follow up were obtained.

Statistical analysis was performed using SPSS version 27.0 to compare clinicopathological features and prognosis between SPNs in patients above 20 and patients under 20 years of age. Chi-square and Fisher’s exact tests were performed. *p*-value < 0.05 was considered statistically significant.

## Results

### Epidemiological data and types of specimens in patients above 20 years of age

During the period of study, 21 cases of SPN were reported in patients above 20 years of age. Ages of patients ranged from 22 to 49 years. Twenty patients (95.2%) were females. In 15 cases (71.4%), pancreatic resection specimens were received. Three cases (14.3%) were received as incisional biopsies while 3 cases (14.3%) were received as blocks for second opinion from other hospitals. Nine out of 21 cases (42.8%) were in the tail of pancreas, 2 cases (9.5%) were located in the body and 1 (4.8%) in the uncinate process. In the 3 cases received as blocks for second opinion, exact location was not known. Of the 15 resection specimens, 8 (53.5%) were distal pancreatectomies, while 7(46.7%) were Whipple resections.

### Tumor size, gross appearance and tumor extent in patients above 20

Tumors ranged from 3.5 to 15 cm in largest dimension with mean size of 7.0 cm. In 14 out of 15 resection specimens (93.3%), tumor was confined to the pancreas and resection margins were clear. Gross appearance was similar to that seen in SPNs under 20 years. Histological features in patients above 20 are summarized in Table 1.

**Table 1:** Histological features of SPNs in patients above 20 years (*n* = 21)

Histological Features	Number of cases	Percentage (%)
Pseudopapillary architecture	21	100%
Solid areas	21	100%
Eosinophilic cytoplasm	16	76.2%
Myxoid stroma	12	57.1%
Hemorrhage	13	61.9%
Infarction	14	66.7%
Fibrosis	10	47.6%
Foamy/ hemosiderin laden macrophages	12	57.1%
Clear cells	15	71.4%
Hyaline globules	16	76.2%
Necrosis	5	23.8%
Cholesterol clefts	5	23.8%
Cystic degeneration	12	57.1%
Microcystic change	11	52.4%
Nuclear grooves	10	47.6%
Mitotic activity	3	14.3%
Atypical cells	3	14.3%
Giant cells	2	9.5%
Calcification	3	14.3%

### Epidemiological data and types of specimens in patients under 20

A total of 29 cases in patients under 20 years of age were included in the study. Ages of the patients ranged from 12 to 19 years. Twenty-eight out of 29 patients (96.5%) were females. In 19 out of 29 cases (65.5%), pancreatic resection specimens were received. In 4 cases (13.8%), incision biopsies were received. The remaining 6 cases (20.7%) were received as blocks for second opinion with primary diagnosis having been made at other institutions. These included 5 incisional biopsies and 1 resection (distal pancreatectomy). In case of the resection specimen, only representative blocks from tumor were received.

Tumor location was tail of the pancreas in 11 cases (37.9%), head of pancreas in 8 cases (27.6%), body in 4 cases (13.8%) and the uncinate process in 1 case (3.5%). In 5 cases (17.2%) which were received as blocks for second opinion, exact location of the tumor was not known. Abdominal pain, mainly centered in epigastrium, was the commonest clinical symptom. Of the 19 resection specimens, 10 (52.6%) were partial (distal) pancreatectomies while 9 (47.4%) were pancreaticoduodenectomies (Whipple resections).

### Tumor size, gross appearance and tumor extent in patients under 20

**Fig. 1:**
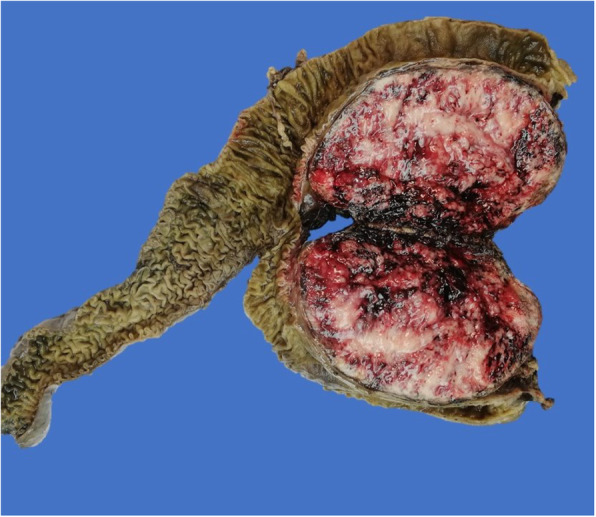
Gross appearance of SPN. Well circumscribed mass in head of pancreas. Cut surface is partly solid with focal hemorrhagic and cystic areas

Tumor size ranged from 3 cm to 14 cm in the largest dimension with a mean size of 9.5 cm. In 17 out of 19 resection specimens (89.5%), tumor was confined to the pancreas and resection margins were negative with distances ranging from 0.2 to 1.5 cm. In 2 cases, tumor extended beyond the pancreas and involved splenic hilum and duodenal wall respectively. Lymph nodes were recovered in 5 out of 19 cases (26.3%). Average number of recovered lymph nodes was 13 per case. Two out of 29 cases (6.9%) had evidence of distant metastases. One of these tumors was located in the tail of the pancreas and metastasized to the liver while the other tumor was located in the head of pancreas and metastasized to the omentum. Grossly, the tumors were nodular, circumscribed, partly or wholly encapsulated lesions with gray white solid cut surfaces. Tumors were firm to friable in consistency and cut surfaces in most cases showed hemorrhagic, infarcted and necrotic areas, foci of cystic degeneration and cavitation (Fig. 1).

### Histological features

**Fig. 2:**
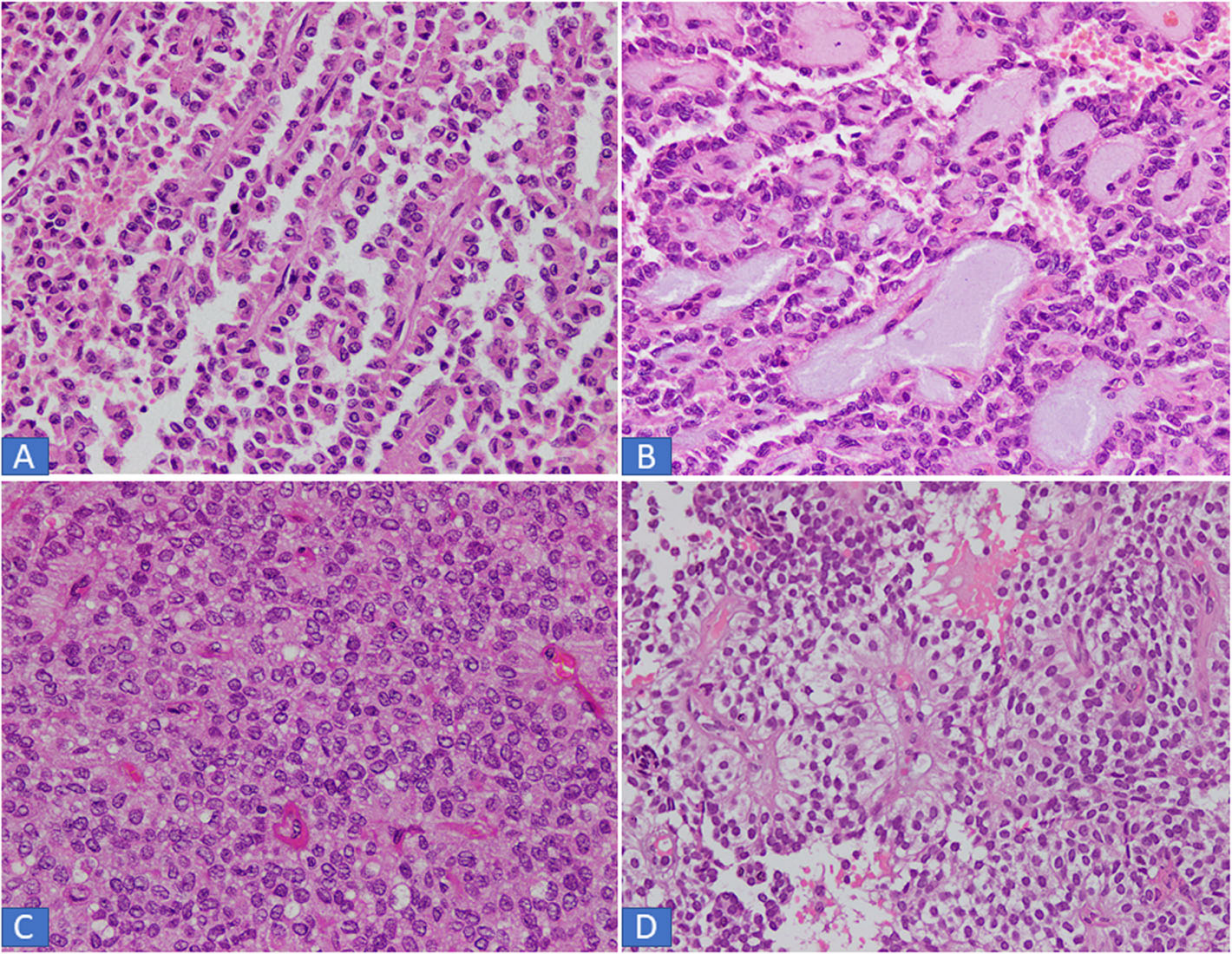
**a** Pseudopapillary structures are a prominent feature in SPN. Tumor cells with eosinophilic cytoplasm are arranged around delicate fibrovascular cores **b** Fibrovascular cores often demonstrate a myxoid stroma. **c** Solid sheets of tumor cells. Some vascular cores can be seen. **d** Cells with clear cytoplasm

**Fig. 3:**
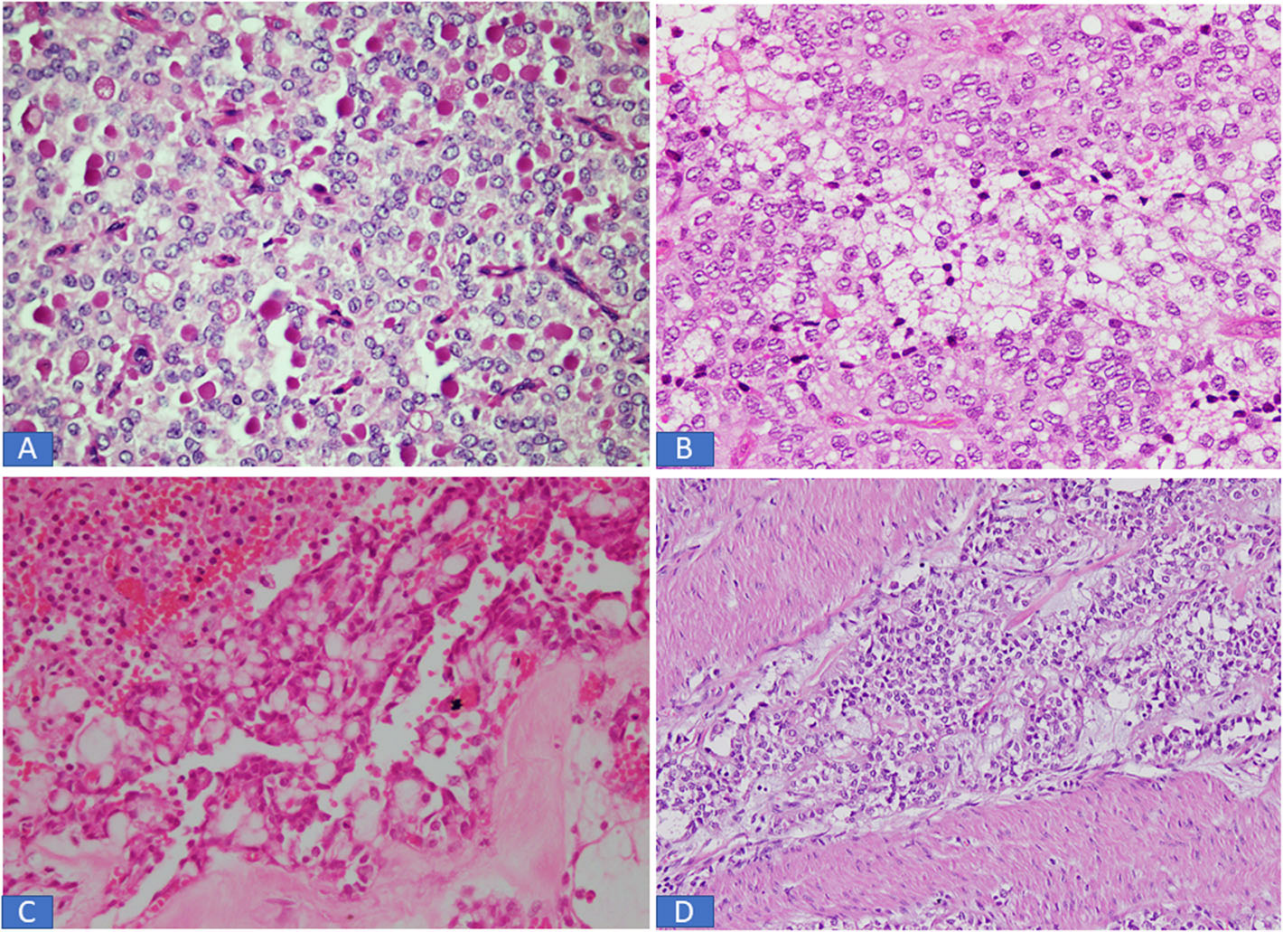
**a** A number of eosinophilic hyaline globules are seen in the cytoplasm of tumor cells **b** Collections of foamy histiocytes are often seen. **c** Focal microcystic areas are not an infrequent feature. **d** A tumor in our series seen involving duodenal wall

A combination of solid and pseudopapillary areas was seen histologically in all 29 cases (Fig. 2a, b, c). Cystic degeneration was seen in 3 cases (10.3%). Cytoplasm was eosinophilic in 23 cases (79.3%), and eosinophilic to clear (Fig. 2d) in 6 cases (20.7%). No significant nuclear atypia or mitotic activity was seen in any of our cases. Nuclear grooves, hyaline globules (Fig. 3a) and foamy histiocytes (Fig. 3b) were seen in 6 (20.7%), 9 (31.1%) and 17 (58.6%) cases respectively. Microcystic change (Fig. 3c) was noted in 7 cases. Involvement of the duodenal wall was seen in one case (Fig. 3d). The histological features of all 29 cases under 20 are summarized in Table 2.

**Table 2:** Histological features of SPN in patients under 20 years of age (*n* = 29)

Histological features	Resection specimens (***n*** = 20)		Incision biopsies (***n*** = 9)	
	Number of cases present	Percentage (%)	Number of cases present	Percentage (%)
Pseudopapillary architecture	20	100%	9	100%
Solid areas	20	100%	9	100%
Eosinophilic cytoplasm	16	80%	7	77.7%
Myxoid stroma	16	80%	7	77.7%
Hemorrhage	15	75%	7	77.7%
Infarction	14	70%	6	66.7%
Fibrosis	14	70%	6	66.7%
Foamy/ hemosiderin laden macrophages	12	60%	5	55.5%
Clear cells	9	45%	4	44.4%
Hyaline globules	6	30%	3	33.3%
Necrosis	8	40%	4	44.4%
Cholesterol clefts	7	35%	3	33.3%
Cystic degeneration	2	10%	1	11.1%
Microcystic change	5	25%	2	22.2%
Nuclear grooves	5	25%	2	22.2%
Mitotic activity	5	25%	3	33.3%
Atypical cells	3	15%	2	22.2%
Giant cells	4	20%	2	22.2%
Calcification	3	15%	1	11.1%

### IHC expression

**Fig. 4:**
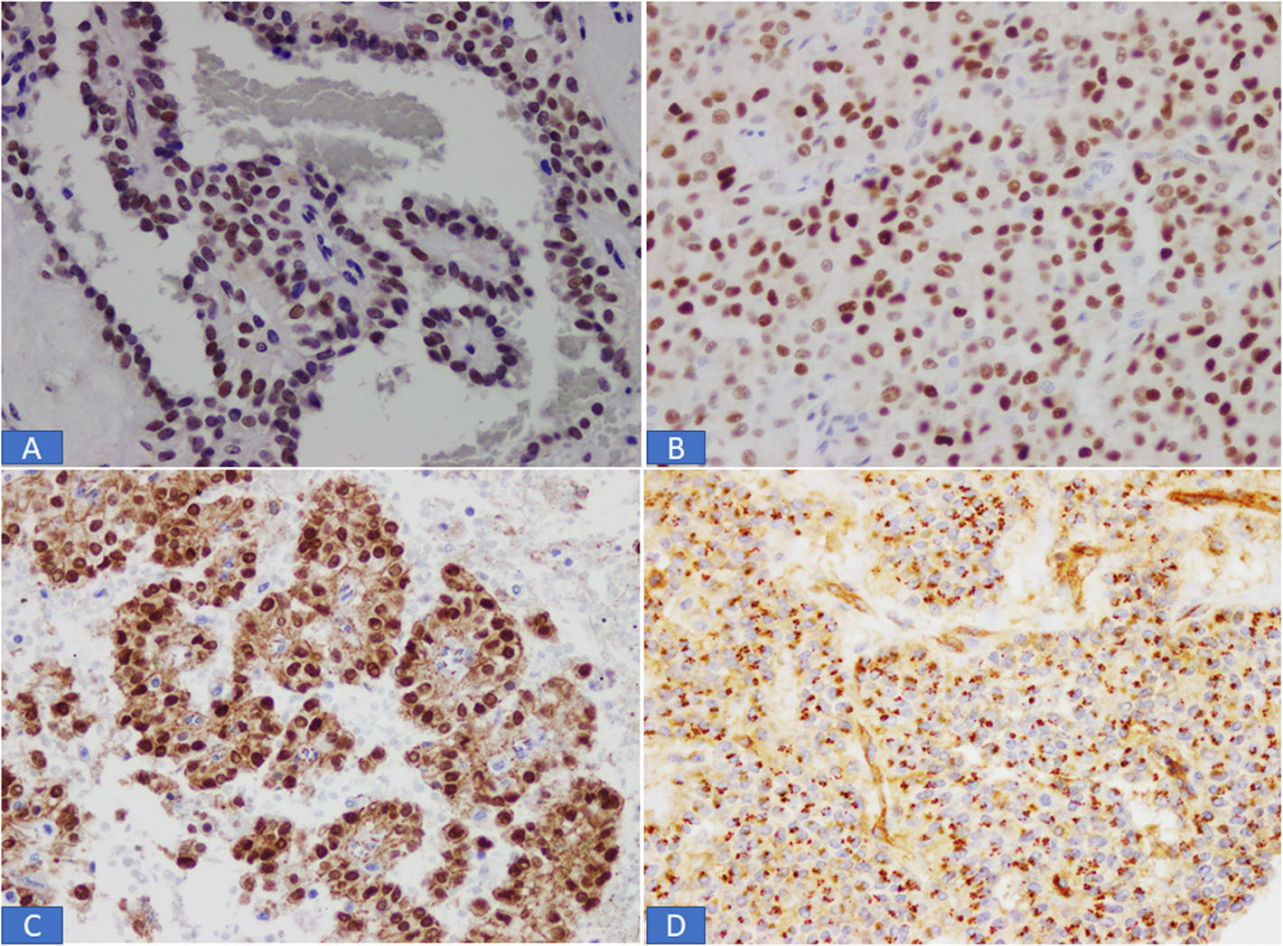
Immunohistochemistry in SPN. **a** Nuclear positivity for TFE3 **b** Diffuse strong nuclear positivity for Cyclin D1 **c** Nuclear positivity for beta catenin **d** Cytoplasmic dot-like positivity for CD99 may demonstrate a unique staining pattern for diagnosis of SPNs

**Fig. 5:**
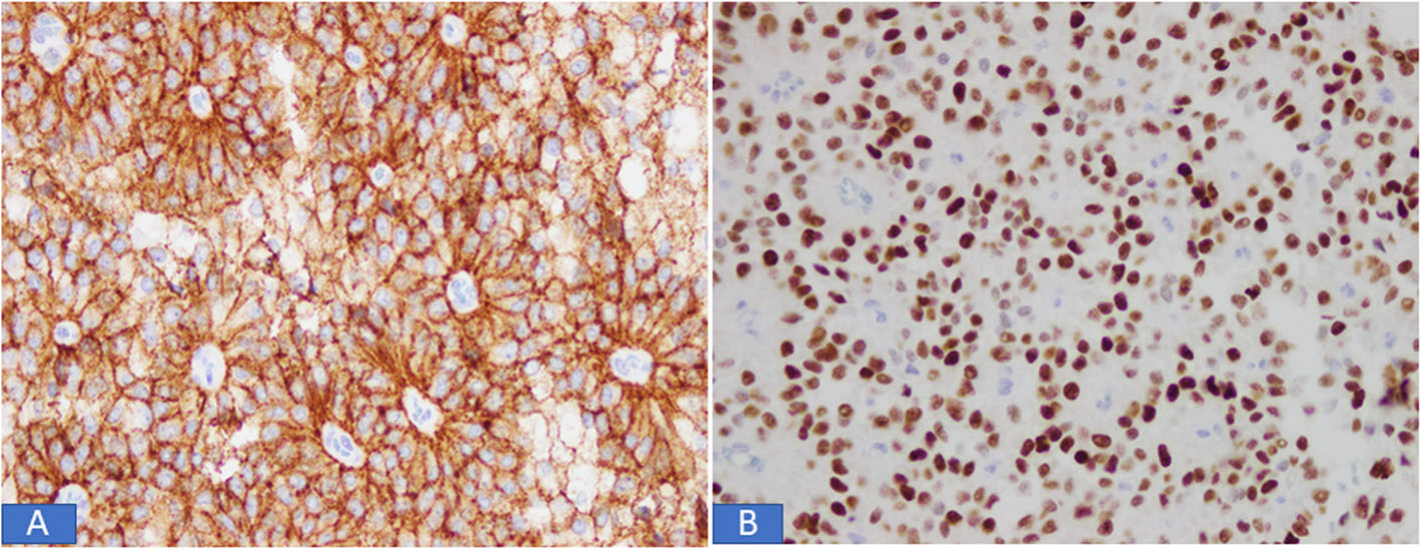
Immunohistochemistry in SPN. **a** Membranous positivity for CD56 and **b** Strong nuclear positivity for PR

IHC stain for TFE3 (Fig. 4a) was performed in 21 cases and demonstrated positivity in 12 cases (57.1%). Cyclin D1 (Fig. 4b) was performed in 13 cases and was positive in 12 (92.3%). Beta catenin (Fig. 4c) was performed in 16 cases and was positive in 15 (93.7%). Cytoplasmic dot like positivity of CD99 (Fig. 4d) was noted in all 6 (100%) cases in which it was performed. CD56 (Fig. 5a) and PR (Fig. 5b) were positive in 19 and 13 cases respectively. The details of all IHC stains performed are shown in Table 3. Clinical follow up was available in 14 out of 29 cases (48.3%). Of these 14 patients, 12 (85.7%) were alive and well without evidence of recurrence and / or metastasis for follow up durations ranging from 5 to 60 months’ post-surgical resection. Median follow up time was 26 months. Two out of 14 patients (14.3%) were alive but developed recurrence and metastases to liver and omentum respectively 41- and 9-months post resection. Six out of 14 patients (42.8%) received on average 3 to 6 cycles of chemotherapy post-surgical resection. The remaining 8 patients did not receive chemotherapy or any other treatment post resection. However, 3 of these 8 patients were scheduled to undergo chemotherapy which was delayed due to the lock down imposed in the wake of the Covid-19 pandemic. These include 1 patient who developed omental metastases 9 months’ post resection and 2 patients with direct involvement of duodenal wall and splenic hilum 5- and 17-months post resection respectively. All 14 patients were being followed up clinically by means of CT scans yearly. The details of follow up are summarized in Table 4.

**Table 3:** Immunohistochemical profile of SPN cases in patients under 20 years of age (*n* = 29)

S. No	Immunohistochemical Antibody	No of cases in which performed	Positive	Type of staining	Negative
1	TFE3	21	12(57.2%)	Nuclear	9(42.8%)
2	Cyclin D1	13	12(92.3%)	Nuclear	1(7.7%)
3	Beta Catenin	16	15(93.8%)	Nuclear	1(6.2%)
4	CD99 (mic2)	6	6(100%)	Cytoplasmic dot-like	–
5	CD10	25	25(100%)	Cytoplasmic	–
6	CD56	19	19(100%)	Membranous	–
7	Progesterone Receptor (PR)	13	13(100%)	Nuclear	–
8	Vimentin	8	8(100%)	Cytoplasmic	–
9	Cytokeratin AE1/AE3	26	19(73.1%)	Cytoplasmic	7(26.9%)
10	Chromogranin A	10	2(20%)	Cytoplasmic	8(80%)
11	Synaptophysin	22	13(59.1%)	Cytoplasmic	9(40.9%)

**Table 4:** Details of follow-up of SPN cases in patients under 20 years of age (*n* = 14)

S. No	Year of resection	Age	Sex	Treatment received	Alive	Recurrence / metastasis	Length of Follow Up
1	2015	19	F	Received chemotherapy	Yes	No	70 months
2	2016	18	M	Received chemotherapy	Yes	No	52 months
3	2016	13	F	No additional treatment received	Yes	No	48 months
4	2016	18	F	No additional treatment received	Yes	No	45 months
5	2017	12	F	Received 3 cycles of chemotherapy	Yes	Liver Metastasis	41 months
6	2017	17	F	Received chemotherapy	Yes	No	39 months
7	2017	17	F	No additional treatment received	Yes	No	30 months
8	2018	15	F	Received chemotherapy	Yes	No	26 months
9	2018	14	F	Received 6 cycles of chemotherapy	Yes	No	22 months
10	2018	18	F	No additional treatment received	Yes	No	19 months
11	2018	14	F	No additional treatment received	Yes	No	17 months
12	2019	19	F	No additional treatment received	Yes	No	14 months
13	2019	15	F	No additional treatment received	Yes	Omental Metastasis	09 months
14	2019	14	F	No additional treatment received	Yes	No	05 months

### Follow up

Follow up in patients above 20 years of age was available in 11 out of 21 cases (52.4%). Follow up period ranged from 3 months to 70 months. 10 (90.9%) patients were alive and well. One patient had developed metastatic disease on initial follow up but was later lost to follow up.

Differences in SPNs in patients above 20 and under 20 years of age in tumors located in tail and head of pancreas were not statistically significant (*p*-value 0.829 for both). However, differences in tumor size were statistically significant (*p*-value 0.0001). No significant statistical difference was found in the above 20 and under 20 group as regard s tumor confined to pancreas (*p*-value 0.881). Similarly, no significant statistical difference in prognosis were found in both age groups (*p*-value 0.987).

Except for tumor size, no statistically significant differences in clinicopathological features or prognosis were observed between SPNs occurring in patients over 20 and those under 20 years of age. Regarding tumor size, larger number of cases in both age groups need to be evaluated.

## Discussion

SPNs should be considered in the differential diagnosis of any solid or partly cystic pancreatic neoplasm in young women under 35 years of age. In our study, tail was the commonest location followed by the head. A 2018 study reported a complication rate of 21% following surgery [23]. SPNs may show extensive necrotic and hemorrhagic areas on cut surface especially when tumors are large. Tumors often have a rubbery consistency and cut surface is characteristically spongy [5]. Of the 19 resection specimens in our series, cut surface in most cases showed areas of hemorrhage, infarction, necrosis, cystic degeneration and cavitation (spongy appearance). In Dubova et al’s series, hemorrhagic foci and blood-filled cavities were seen in 40% cases [9].

Direct splenic invasion can occur in pancreatic tail SPNs [24]. In our series, 1 case located in the tail demonstrated direct splenic invasion. Histologically all 29 cases in our series showed areas with solid sheets of tumor cells and other areas composed of pseudopapillary structures. Solid areas are predominantly found near the capsule of the tumor while pseudopapillae are more common in the central part of the tumor. Tumor cells were poorly cohesive and uniformly arranged around delicate fibrovascular stalks [9]. Tumor cells in our cases were round and monomorphic with oval, frequently grooved nuclei, peripheral nucleoli and pale to clear cytoplasm. Hyaline globules are seen in some cases. These constitute a common and typical feature of SPNs, although they are not specific to these tumors [5, 27]. Cholesterol clefts were also seen in multiple cases. Other histologic features which are evaluated were based on the histologic features described in the 5th edition of WHO Classification of Digestive Tumors and other studies and other studies included myxoid stroma, calcification, aggregates of foamy histiocytes, eosinophilic bodies, multinucleated cells, and clear cells. The results are shown in Table 1. Except for clear cells, none of the other histologic features are significantly associated with aggressive behavior. Clear cells, when present, may be a possible prognostic indicator for the presence of perineural invasion which in turn is a predictive parameter associated with aggressive behavior in these neoplasms [28]. Areas of hemorrhage and infarction were seen in 75.9 and 72.4% of our cases, respectively. Hemorrhagic areas are more common in large tumors. A study demonstrated microcystic pattern in almost 30% SPNs (microcystic SPNs) which may lead to confusion with microcystic pancreatic neoplasms. The study found that clear cell change, hyalinized stroma and hemorrhage were significantly more common in microcystic than conventional SPNs and that microcystic SPNs were much less likely to express IHC markers CD10 and CD56 [29]. We observed microcystic pattern in 24.1% of our cases (Table 1). SPNs demonstrate little or no obvious nuclear atypia and mitoses are rare. A recent study found 0 to 6 mitoses per 20 HPFs. MIB-1 (KI-67) proliferative index was very low [5]. Nuclear atypia and mitotic activity were insignificant in our cases.

The results of IHC staining in our cases are shown in Table 2. Published literature has shown that almost all SPNs demonstrate positivity for vimentin, CD10, CD56, CD99, and alpha- 1 antitrypsin [10, 21, 30–32]. Perinuclear dot like staining for CD99 constitutes a unique staining pattern for diagnosing pancreatic SPNs [5, 7, 32, 33]. Numerous studies over the years have also documented the role of IHC stains such as beta catenin, Cyclin D-1, FLI-1 and E- Cadherin in SPNs [5, 7, 9, 34]. A number of studies including proteomic profiling studies have underlined the importance of disrupted WNT/beta catenin signaling pathways with concomitant cyclin D1 overexpression in the development of pancreatic SPNs. SPNs consistently demonstrate B- catenin mutation with activation of WNT- signaling pathway and resultant overexpression of Cyclin D-1. Cyclin D1, FLI -1, CD56 and PR which are all expressed in SPNs are all localized to chromosome 11 q [5, 35–38]. Beta catenin was present in 16 cases, 1 out of these 16 cases did not show beta catenin expression. However, this case showed the classic histologic features of SPN and demonstrated positivity for IHC stains of CD10, Cyclin D1, CD56, and CD99. Owing to negativity for beta catenin, this case was reviewed in the Intradepartmental Consultation Conference attended by all consultants in the department and a consensus was the consultants that it should be reported as SPN in spite of negativity for beta catenin.

SPNs have been shown to harbor recurrent somatic pathogenic variants in the beta catenin gene, CTNNB1 and these contribute to the pathogenesis of these tumors via the WNT signaling pathway. The activated WNT- signaling pathway is disrupted due to beta catenin mutation [35]. Free beta catenin regulates the WNT pathway by undergoing rapid degeneration. Mutated beta catenin does not undergo degradation resulting in disruption of the WNT pathway. Beta catenin also normally play a role in the coupling of cadherin to the cytoskeleton. E-Cadherin is a member of the transmembrane glycoprotein family which facilitates calcium mediated intercellular adhesion. Mutated beta catenin also causes mutations in E-cadherin gene resulting in abnormal expression of E- cadherin which can be confirmed by IHC. Mutations in E- cadherin gene lead to disturbances in cell adhesion in SPNs and lead to formation of pseudopapillary structures [9, 10, 34]. Nuclear expression of Cyclin D-1 and E-cadherin is seen in 70 to 100% SPNs. Similarly, beta catenin expression is seen in the large majority of SPNs [10, 15, 18, 27, 34]. The nuclear labeling of beta catenin in SPNs helps in differentiation from the membranous labeling seen in Pancreatic Neuroendocrine Tumors (Pan NETs) [15, 18].

It is clear from the above discussion that practically all SPNs are positive for vimentin, beta catenin, cyclin D-1, alpha-1 antitrypsin and CD56 and are typically negative for E-cadherin [5, 10, 29, 33, 39]. PR negativity in SPNs is associated with worse prognosis. Most cases demonstrated positivity for PR. PR was performed in 13 of our cases and positivity was seen in all cases. Negative PR result in SPNs is significantly associated with poorer disease-free survival (DFS) and disease specific survival (DSS). Thus, negative PR staining on IHC is an independent poor prognostic factor and appears to have a role in predicting adverse outcome [8, 10, 39].

In recent years, several new IHC antibodies have become available which are very valuable in the correct diagnosis of SPNs and in differentiating them from other pancreatic neoplasms. The most important of these is TFE3. As discussed above, aberrant WNT signaling is a hall mark of these tumors. TFE3 plays a critical role in the activation and regulation of the WNT pathway and has been shown to be implicated in SPN. Almost 95% SPNs display moderate to intense nuclear accumulation and expression of TFE3. On the other hand, about 15 to 25% Pan NETs, ductal adenocarcinomas and pancreatic neuroendocrine carcinomas respectively show positivity for TFE3. Thus, TFE3 can be useful along with beta catenin as a diagnostic marker for SPN and in differentiating it from other pancreatic neoplasms. Similarly, SOX proteins are key modulators of the WNT/ beta catenin signaling pathway. Recent RNA microarray and gene regulatory network analyses have shown that SOX11 mRNA is consistently increased in SPNs but not in Pan NETs or the normal pancreas. Harrison et al. analyzed the IHC expression of TFE3, SOX 11 and beta catenin in 31 cases of surgically resected SPNs using Pan NETs, acinar cell carcinomas and pancreatoblastomas as controls. Positivity for TFE3 was seen in 30 out of 31 cases (96.8%). Nuclear positivity for SOX-11 was seen in all 31 SPNs and in 5 out of 31 control tumors. Nuclear positivity for beta catenin was noted in all 31 SPNs and 4 control tumors. The combination of these three markers can be used clinically as a diagnostic IHC panel in distinguishing SPNs in indeterminate cases from other pancreatic tumors which may mimic them histologically. SOX11 and TFE3 can be useful as diagnostic markers for SPNs in fine needle aspiration (FNA) biopsies as well. These markers are useful as diagnostic markers for distinguishing SPNs from their cytologic mimics. Kim et al. also investigated the role of TFE3, LEF1 (lymphoid enhancer binding factor 1), Androgen Receptor (AR) and beta catenin in pancreatic SPNs. Positivity for TFE3 was seen in 68 out of 91 cases (74.7%). They reported diffuse nuclear expression of beta catenin as a putative diagnostic feature of SPN in almost 99% cases. LEF1 and AR were also expressed in majority of SPNs, while pancreatic ductal adenocarcinomas and pan NETs showed no expression. A combined IHC panel of beta catenin, LEF1 and TFE3 resulted in a sensitivity and specificity of 100 and 91.9% in distinguishing SPNs from ductal adenocarcinomas and Pan NETs. Thus, SOX11 and other transcription factors are important in the diagnosis of SPNs and in distinguishing them from Pan NETs and pancreatic ductal adenocarcinomas. The above discussion makes it clear that the combination of several IHC markers ensures accurate diagnosis of SPNs and reduces the chances of misdiagnosis [15, 18, 37, 39].

A recent study by Walters et al. compared pediatric and adult SPNs and found that there were similarities in demographics, tumor characteristics and treatment modalities. However, survival was shown to better in children [40].

Although SPNs are considered to have low grade malignant potential, majority of cases are cured following complete surgical resection and 5-year survival rates are excellent [41–44]. About 10 to 15% of cases may demonstrate malignant behavior characterized by tumor recurrence, invasion of adjacent organs and/ or metastasis [45–47]. Surprisingly, one recent study showed histologic features consistent with poor prognosis in large majority of SPNs [48]. However, even tumors with malignant features are associated with excellent prognosis and patients can be expected to have long survival following aggressive surgery [49]. A French study on pediatric SPNs by Irtan et al. also showed tumor recurrence in pediatric SPNs also constitutes a rare and late event and does not undermine overall survival. Complete surgical resection appears to be the best option for recurrent tumors in the pediatric age group as well. Enbloc resection without formal lymphadenectomy is preferred and attempts should be made even to resect distant metastases when present [50, 51].

Six out of 14 patients in our series, in whom follow up was available, received chemotherapy post-surgery. Another 3 recent cases (1 with omental metastases and 2 with direct extension to duodenum and spleen respectively) were scheduled to receive chemotherapy which was delayed due to lockdowns imposed in the wake of the Covid-19 pandemic. Questions can justifiably be raised as to why 5 young patients whose tumors were confined to the pancreas and were adequately resected were given chemotherapy post resection. The possible explanation is that many oncologists in our country (especially in smaller cities) have little knowledge of these rare neoplasms and believe that being low grade malignancies which can sometimes invade adjacent organs and metastasize should be treated aggressively to achieve good long term prognosis in young patients. In presence of radical resection with negative margins, no adjuvant oncological treatment is usually indicated in SPNs as rate of recurrence is very low [17, 25]. Unresectable tumors and metastases occurring within 36 months are independent variables in predicting survival. Completely resected tumors, even those showing aggressive gross and morphologic features have excellent prognosis and patients can still survive for more than 10 years [52, 53]. SPNs have in the past been considered “benign” or “borderline tumors” but recent molecular evidence demonstrating alterations in cancer associated genes and the ability of these tumors to metastasize have confirmed their malignant nature [54]. Long term follow-up is mandatory in order to detect delayed metastases [38].

## Conclusions

Clinico-epidemiological, morphologic and IHC findings of these rare tumors in patients under 20 years of age are presented. The importance of a detailed IHC panel in differentiating these tumors from histologic mimics and reaching an accurate diagnosis is highlighted. Factors affecting prognosis and predictive of aggressive behavior (histological, IHC, clinical, and type of surgery) are discussed. A detailed review of published literature is presented to provide readers a comprehensive yet succinct account of these rare pancreatic neoplasms. Although many studies on SPNs have been published, surgeons, oncologists and even pathologists in this part of the world are often not aware of these rare tumors leading to inaccuracies and delays in diagnosis. In addition, this paper focusses on the interesting observation that the majority of SPNs diagnosed in our department during study period occurred in patients under 20 years of age (29 versus 21 in patients over 20). However, clinico-epidemiological, morphologic and prognostic features were similar in both age groups. New antibodies such as TFE3 and prognostic importance of PR in SPNs are also discussed. We believe that this is a very interesting and helpful study for the clinicians as well as the pathologists. Possibility of SPNs should always be considered in case of pancreatic neoplasms occurring in patients under 20 years of age as well.

## Data Availability

Data and materials of this work are available from the corresponding author on reasonable request.

## Abbreviations

SPN: Solid Pseudopapillary Neoplasm
PR: Progesterone Receptor
WHO: World Health Organization
IHC: Immunohistochemical
CT: Computed Tomography
PAS: Periodic Acid Schiff
Pan NET: Pancreatic Neuroendocrine Tumor
DFS: Disease-free Survival
DSS: Disease specific Survival
FNA: Fine Needle Aspiration
AR: Androgen Receptor
